# IgG and IgM responses to the *Plasmodium falciparum* asexual stage antigens reflect respectively protection against malaria during pregnancy and infanthood

**DOI:** 10.1186/s12936-024-04970-7

**Published:** 2024-05-19

**Authors:** Mahugnon L. Erasme Gbaguidi, Rafiou Adamou, Sofie Edslev, Anita Hansen, Nadia D. Domingo, Celia Dechavanne, Achille Massougbodji, André Garcia, Michael Theisen, Jacqueline Milet, Eduardo A. Donadi, David Courtin

**Affiliations:** 1 Division of Clinical Immunology, Department of Medicine, Ribeirão Preto Medical School, University of São Paulo, São Paulo, Brazil; 2 IRD, MERIT, Université Paris Cité, 75006 Paris, France; 3 Centre d’Etude Et de Recherche Sur Les Pathologies Associées À La Grossesse Et À L’Enfance, Cotonou, Bénin; 4 Institut de Recherche Clinique du Bénin, Abomey-Calavi, Benin; 5 Centre for Medical Parasitology at Department of International Health, Immunology and Microbiology, University of Copenhagen, Rigshospitalet, Copenhagen, Denmark; 6 Department of Infectious Diseases, Copenhagen University Hospital, Copenhagen, Denmark; 7 Department for Congenital Disorders, Statens Serum Institut, Copenhagen, Denmark

**Keywords:** IgG, IgM, Asexual stage, *Plasmodium falciparum*, Pregnancy, Infancy

## Abstract

**Background:**

*Plasmodium falciparum* malaria is a public health issue mostly seen in tropical countries. Until now, there is no effective malaria vaccine against antigens specific to the blood-stage of *P. falciparum* infection. Because the pathogenesis of malarial disease results from blood-stage infection, it is essential to identify the most promising blood-stage vaccine candidate antigens under natural exposure to malaria infection.

**Methods:**

A cohort of 400 pregnant women and their infants was implemented in South Benin. An active and passive protocol of malaria surveillance was established during pregnancy and infancy to precisely ascertain malaria infections during the follow-up. Twenty-eight antibody (Ab) responses specific to seven malaria candidate vaccine antigens were repeatedly quantified during pregnancy (3 time points) and infancy (6 time points) in order to study the Ab kinetics and their protective role.

Abs were quantified by ELISA and logistic, linear and cox-proportional hazard model were performed to analyse the associations between Ab responses and protection against malaria in mothers and infants, taking into account socio-economic factors and for infants an environmental risk of exposure.

**Results:**

The levels of IgM against MSP1, MSP2 and MSP3 showed an early protective response against the onset of symptomatic malaria infections starting from the 18th month of life, whereas no association was found for IgG responses during infancy. In women, some IgG responses tend to be associated with a protection against malaria risk along pregnancy and at delivery, among them IgG3 against GLURP-R0 and IgG2 against MSP1**.**

**Conclusion:**

The main finding suggests that IgM should be considered in vaccine designs during infanthood. Investigation of the functional role played by IgM in malaria protection needs further attention.

**Supplementary Information:**

The online version contains supplementary material available at 10.1186/s12936-024-04970-7.

## Background

*Plasmodium falciparum* malaria is transmitted by the bite of the *Anopheles* mosquito, representing a major endemic problem in sub-Saharan African countries. According to the 2022 World Health Organization (WHO) report, there are more than 249 million cases of malaria and approximately 608,000 deaths worldwide, and pregnant women and children younger than five years are the population groups at higher risk [1]. For many decades, malaria control has been based on vector control strategies, primarily including long lasting insecticide treated nets and intra-domiciliary insecticide applications, as well as on the drug administration based on artemisinin-based combined therapy [2–4]. Notwithstanding, these strategies have been limited by the development of mosquito resistance to insecticides and parasite resistance to the available treatment [5–7], requiring the search for new tools such as vaccines to alleviate malaria attacks and complications.

The RTS,S, Mosquirix™ vaccine, based on circumsporozoite protein (CSP) antigen, is the most advanced malaria vaccine. The RTS,S vaccine may increase malaria protection up to 70% in children aged from 5 to 17 months when associated with intermittent preventive treatment [8]. However, the RTS,S vaccine has shown a relatively low efficacy (32.1% to 37.6%) against severe malaria in children aged from 3 to 5 years, even when used in frequent injections [9]. To improve the effectiveness of the malaria vaccine, it is important to understand the protective mechanisms by exploring the efficacy of candidate vaccine antigens. Asexual blood-stage *P. falciparum* are involved in the development of clinical symptoms and merozoite surface proteins (MSP) 1, MSP2 and MSP3, apical membrane antigen-1 (AMA1), and glutamate-rich protein (GLURP) are potential targets that may help on malaria control [10]. Identification of the best candidate vaccine antigens under natural condition of malaria exposure could help on the design of vaccines including antigens derived from different stages of *P. falciparum* life cycle.

Several lines of evidence have shown the role of antibodies (Abs) on malaria protection: i) Abs from immune adults had an effective protective role against *P. falciparum* when transferred to non-immune young children suffering from uncomplicated malaria [11], ii) *in-vitro* experiments such as growth inhibition assay, antibody-dependent cellular inhibition, antibody-dependent respiratory burst, and opsonized phagocytosis have shown an association between Ab level and clinical protection against malaria in infected children [12, 13], iii) the presence of a histidine residue (H435) at the IgG3 binding domain to FcRn increases the transplacental transfer and the half-life of this specific anti-merozoite Ab in infants [14], iv) the development of IgG1 and IgG3 cytophilic Abs against merozoite in infants or in children has been associated with the control of malaria infection [15, 16], and v) besides IgG, IgM Abs against *P. falciparum* asexual stage antigens are also associated with protection against malaria infection in mothers and their infants [17], inhibiting *P. falciparum* parasite development by complement-dependent cytotoxicity during malaria in children and adults [18]. Taken together, these results show the complex relationship regarding the development of protective Abs against *P. falciparum* asexual stage antigens in pregnant women and in the newborn.

The protective role of IgG and IgM Abs against a panel of asexual stage antigens was assessed using a comprehensive Beninese cohort of pregnant women and their infants together with entomological, socio-environmental, epidemiological, and parasitological data, aiming to identify the most effective vaccine antigens for these populations.

## Methods

### Study area and population

The present study is part of the “*Tolérance Immunitaire et Paludisme*” (TOLIMMUNPAL) project, conducted from January 2010 to June 2013 in the Beninese district of Allada (Sékou and Attogon health centres), a Southern semi-rural area, where the *P. falciparum* malaria is hyperendemic with an average of 20.5 infected anopheles bites/person/year [19]. The TOLIMMUNPAL project is a continuation of two other studies: i) a large project called “Malaria in Pregnancy Preventive Alternative Drugs” (MiPPAD) and ii) the “Anemia in Pregnancy: Etiology and Consequences” (APEC) study. MiPPAD was a clinical trial encompassing 4,749 pregnant women from Benin, Gabon, Mozambique, and Tanzania [20, 21], of whom, 1,005 Beninese women participated. The study evaluated the efficacy and safety of intermittent preventive treatment of malaria in pregnancy (IPTp) with three arms: a single dose of Mefloquine (MQFD for Full Dose of Mefloquine), a split dose of Mefloquine over 2 days (MQSD for Split-Dose of Mefloquine) and a dose of Sulfadoxine-Pyrimethamine (SP). Four-hundreds of these Beninese pregnant women participated in the APEC study. The study was designed to follow-up the consequences of maternal anaemia on clinical and biological features of their children along the first year of life [22, 23]. The participants of this study were recruited from April 9th 2010 to June 14th 2011. In the TOLIMMUNPAL project, the infants of the APEC study were followed-up for an additional year until the age of 24 months to assess the clinical, genetic, immunological, and environmental determinants of malaria [24]. The study population encompassed only HIV-negative pregnant women and their offspring. Twin pregnancies, pregnancies complicated by stillbirth, or fetal abnormalities were excluded.

### Mother–child follow-up

The details of studied sample and the procedures of data collection are described elsewhere [22] [24]. Briefly, 400 pregnant women, receiving their first IPTp at the first antenatal visit (ANV1, at gestational age ≤ 28 weeks), were followed-up until delivery. They received the second dose of IPTp at the second antenatal visit (ANV2, at least one month later). At both ANVs and at delivery, women underwent a clinical examination when thick blood smears (TBS) were collected. Throughout the follow-up, malaria infections were detected with a passive surveillance: women were invited to attend the health centre in case of any complaints. When an axillary temperature was greater than or equal to 37.5 °C, a TBS was performed. Clinical data (gravidity, gestational age), sociodemographic data (age, schooling, and ethnic group) as well as socioeconomic data (possession of a bicycle, a refrigerator, a television, having electricity, having a gainful activity) were collected. At birth, newborn’s sex and weight were recorded and gestational age was evaluated using the Ballard score [25]. From birth to 12 months of age, infants were examined at three scheduled visits at 6, 9 and 12 months. During this period, symptomatic malaria cases were also detected at the health centre. Then, from 12 to 24 months, children were actively followed-up with a home visit twice a month. In case of axillary temperature greater than or equal to 37.5 °C (or a history of fever in the preceding 24 h), a rapid diagnosis test (RDT) and a TBS were performed. A scheduled home visit was planned once a month. During each scheduled visit, infants were clinically examined, a TBS was performed to identify asymptomatic infections, and information about the use of mosquito nets was collected. Throughout follow-up, women were invited to bring their children to the health centre for any health concern, where all medications were prescribed free of charge. According to the recommendations of the Beninese National Malaria Control Programme, all malaria cases were treated with artemether-lumefantrine.

### Sample collection

From the pregnant women, 5 mL peripheral blood sample (EDTA) from each time point (at ANV1, ANV2 and Delivery) and 5 mL of the cord blood were collected for further analyses. The plasmas were collected for the measurement of Ab levels. The diagnosis of placental malaria (PM) was performed using a placental blood smear stained with Giemsa [20]. From the newborn, 5 mL peripheral blood (EDTA) were drawn during scheduled visits at 6, 9, 12, 18, and 24 months of age and plasma was collected for the measurement of Ab levels.

***Ethical committee.***

The institutional review boards of the Comité Consultatif de Déontologie et d'Éthique from the Institut de Recherche pour le Développement (France), the Ethic Committee of the Faculty of Health Sciences (University of Abomey-Calavi, Benin) and the Human Research Ethics Committee of the University Hospital of the Ribeirão Preto Medical School of the University of São Paulo (Brazil) approved all study protocols included in this paper. Before inclusion, the study was explained and written informed consent of women was obtained in the presence of a witness, with thumbprints provided if women could not read and/or write.

### Prediction of the environmental risk by entomological and geographical variables

Mosquito exposure was evaluated using a previous developed predictive model taking into account entomological (mosquito captures), geographical (village, distance from a watercourse), climatic (season, rainfall between two monthly visits, number of rainy days in the 10 days before the visit), and environmental data (type of roof and wall of the house, characteristics of its immediate surroundings, normalized difference vegetation index) collected throughout the follow-up [26]. The mosquitoes were caught during two consecutive nights by month in the house of 180 out of the 400 infant's bedrooms, using Centre for Disease Control light traps, from April 2011 to February 2013. The selection of 180 bedrooms was made on the basis of homogeneous geographical distribution. This predictive model allowed the estimation a quantitative risk of mosquito exposure for each child once a month, at the date of each scheduled visit. A high level of this variable indicates a high mosquito exposure.

### Measurement of antibody concentrations

IgG1, IgG2, IgG3, and IgM concentrations against candidate *P. falciparum* asexual vaccine antigens (AMA-1_25–545_, MSP-1_19_, MSP2-3D7, MSP2-FC27, MSP-3_212–380_, GLURP-R0_25–514_ and GLURP-R2_705–1178_) were measured using ELISA, as proposed by the African Malaria Network Trust (AMANET), using plasma collected at ANV1, ANV2, and delivery for the mothers and at birth, 6, 9, 12, 18 and 24 months of age for the infants. The recombinant AMA1 antigen (residues 25–545, obtained through *Pichia pastoris*) was provided by the Biomedical Primate Research Centre (Rijswijk, The Netherlands). The MSP-1_19_ antigen was produced in Paris by Institut Pasteur through a Baculovirus/insect cell system and was composed by two peptides (amino acids 1–43 and 1615–1723, Uganda-Palo-Alto strain). The MSP2 antigens (3D7 and FC27, without the secretion signal and the GPI anchor) were produced at the University of La Trobe (Melbourne, Australia). MSP3 (amino acids 212–380, F32 strain), GLURP-R0 (amino acids 25–514, F32 strain) and GLURP-R2 (amino acids 705–1178, F32 strain) were obtained through *Escherichia coli* by the Statens Serum Institute (Copenhagen, Denmark). The selection of these antigens including in AMANET network is in order to use a standardized protocol to facilitate result comparison between different teams.

Briefly, the 96 well plates (Nunc MaxiSorp, Thermo Fisher Scientific, Roskilde, Denmark) were coated with 1 μg/mL of the vaccine candidate antigen in PBS and incubated at 4◦C overnight. Plates were washed using buffer (PBS with 0.1% Tween-20 and 0.5 M NaCl) and the reaction was blocked for one hour using 3% powdered-milk with 0.1% Tween 20 and PBS. Diluted plasma samples (1X PBS with 1% powdered-milk, 0.1% Tween-20 and 0.2% NaN3) were added, followed by two hours of incubation at room temperature. Maternal samples and cord blood were diluted 1/100 for all antigens, except AMA-1, for which the dilution was 1/2000, while in children plasma samples, the dilution was 1/50 for all antigens, except AMA-1, which was 1/1000. After washing, the peroxidase conjugated sheep anti-human IgG1 (The Binding Site, 330372A) in dilution buffer (1:5000), sheep anti-human IgG2 (The Binding Site, 326,187) in dilution buffer (1:2000), sheep anti-human IgG3 (The Binding site, 327,290) in dilution buffer (1:5000), goat anti-human IgM (The Binding Site, 1111791C) in dilution buffer (1:3000) were added to the plate and incubated for one hour at room temperature. The reaction was revealed with the use of TMB (3, 30, 5, 50 tetramethylbenzidine, Kem-En-Tec, Copenhagen, Denmark) as substrate and stopped with 0.2 M H2SO4.

Purified human IgG1, IgG2, and IgM proteins with well-known concentrations (pg/mL) served as standards, starting from 500; 250; 125; 62.5; 31.3; 15.6; 7.8 to 3.9 pg/mL, while for IgG3 the concentrations started from 100; 50; 25; 12.5; 6.25; 3.13; 1.56 to 0.78 pg/mL. ODs were measured at 450 nm and analysed with ADAMSEL FLP b039 software (http://www.emvda.org/portfolio/project-index/optimalvac-project completed) for determination of Ab concentrations. The samples were re-evaluated when the coefficient of variation between the discordant duplicates exceeded 15%.

### Statistical analyses

All statistical analyses were performed using the R [27] and the Rstudio v 1.0.153 softwares [28]. Prior to analyses, data regarding Ab concentrations were log-transformed except for the analysis of Ab transfer, which was evaluated as the ratio between the neonatal and maternal Ab concentrations. After log-transformation, Ab concentrations exhibited a Gaussian distribution. For all analyses, the threshold value for statistical significance was set at p < 0.05.

### Antibody dynamics analyses

Anti-asexual stage Ab concentrations were analysed considering different time points and different settings of the follow-up, including the following comparisons: i) At ANV1, ANV2, and delivery to evaluate the dynamics of Ab response along gestation, ii) At delivery (peripheral blood for the mother and cord blood for the newborn) to evaluate the transfer of Ab from mother to the newborn, iii) At different time points (at 6, 9, 12, 18 and 24 months) to evaluate the production of Abs during the first two-years of life. Paired t-tests were used to perform a comparison of Ab concentrations between mothers and infants at delivery, whereas a linear mixed model was used to make a pairwise comparison of Ab concentration at several time points during pregnancy and during infancy. Using the packages lme4 [29] and emmeans [30], the linear mixed model allowed to determine the differences between time-points, and the significance of the pairwise differences was evaluated with the Tukey method. The correlations between Ab concentrations of the peripheral blood (mother) and the cord blood (newborn) were also performed using the Pearson correlation coefficient.

### Analyses of maternal antibody transfer

The transfer of maternal Abs was estimated by the ratio between the Ab concentrations observed in the cord blood (newborn) and in the peripheral blood (mother). Non-parametric tests were used to compare the Ab transfer between IgG subclasses for each antigen and between antigens for each subclass. In order to identify the factors influencing the maternal Ab transfer, a multiple linear regression was used after exclusion of atypical values exhibiting a ratio > 2. The following factors that may influence the maternal Ab transfer were: the age of the mother (in four 5-years groups), ethnic group, Ballard score, gravidity (primigravidae *versus* multigravidae), the concentrations of anti-asexual stage Abs at ANV2 and at delivery, the transmission season at ANV2, the presence of a malaria infection during pregnancy, the number of infections during pregnancy, the presence of an infection between ANV2 and delivery, placental malaria, and the arm of IPTp clinical trial. Univariate analyses were performed for each of the 28 Ab responses studied (three subclasses of IgG and IgM measured against seven antigens). Afterwards, a multivariate model was defined by type of Ab response (IgG1, IgG2, and IgG3). Factors included in multivariate models were those exhibiting a *p* < 0.05 in the univariate analyses for at least one of the seven antigen responses. Multicolinearity in the multivariate model was checked using the variance inflation factor (VIF).

### The protection of antibodies against malaria infection

The protective association conferred by antibodies is defined here as a correlation between the control of malaria susceptibility risk (delay in the occurrence of first *P. falciparum* infection, decrease in episodes of malaria over a period of time or placental malaria) and the concentration of specific antibody responses. In all analyses, Ab concentrations were considered as a quantitative variable. The effect of the concentration of each Ab response was tested in a multivariate model including the covariates associated with the risk of malaria infections. The covariates considered in the multivariate analyses and the statistical methods used for these analyses are detailed in Additional file 1. Environmental risk was taken into account in the analysis of the protective role of antibodies in mothers and infants, but in different manner. For mothers, the analyses were adjusted for the season of transmission at ANV2, while for infants, the analyses were adjusted for the risk of environmental exposure, as described above, and the season of transmission (rainy season of 2010, 2011 and 2012).

#### Protection of antibodies against malaria infection during pregnancy

The protective role of maternal Abs during pregnancy was evaluated against peripheral infections, between ANV2 and delivery, and against placental infection detected at delivery. To assess protection against peripheral malaria infections, the number of days after ANV2 until reinfection was the dependent variable in the model. A Cox proportional hazards regression model was used to evaluate the effect of Ab concentrations in *P. falciparum* reinfection. The R package survival [31] was used to perform Cox regressions and to check the proportional hazards assumption. The protective role of maternal Abs was assessed at ANV2, because at this time: i) all women had received a second dose of IPTp as part of the clinical trial, abrogating the influence of a putative ongoing malaria infection, and ii) more infections occurred between ANV2 and delivery when compared to ANV1 and ANV2. To assess whether concentrations of maternal Abs protect against placental infection (a binary outcome), a logistic regression was used.

#### Protection of maternal antibodies in infants

Malaria infections were classified as symptomatic or asymptomatic. A symptomatic infection was defined as the presence of fever (or a history of fever) and a positive RDT and/or a positive TBS. An asymptomatic infection was defined as a positive TBS without fever (or a history of fever) and without the diagnosis of a symptomatic malaria episode within the next three days. The effect of Ab response concentrations was evaluated on the time to occurrence of the first malaria infection (symptomatic or asymptomatic), using a Cox-proportional hazards regression model. In addition, the protective effect of maternal Abs was analysed using a negative binomial regression, counting the number of symptomatic malaria episodes up to the age of 18 months and counting the number of symptomatic episodes on the entire follow-up. After each malaria episode, an exclusion period of 14 days was defined because infants were not at risk due to the anti-malarial treatment.

#### Protection of infant antibodies at the age of 12 and 18 months

The protective effect of Ab concentrations, detected at 12 and 18 months of age, was evaluated analysing the time to infection during the six-month periods after blood sampling using a Cox proportional hazards regression model. Thus analyses were performed between 12 and 18 months (for blood collection at the age of 12 months), and between 18 and 24 months (for blood collection at the age of 18 months). Children harbouring malaria infection on the day of blood collection or 14 days before were not included in the analysis to avoid influence on the immunological data.

The protective role of infant’ Abs was assessed in different subgroups: 1) malaria exposed infants; 2) exposed infants with at least one malaria infection before blood collection, and 3) exposed infants with one recent malaria infection (in the last three months). Malaria exposed infants were defined as those experiencing at least two malaria infections during the entire follow-up [32]. The Ab protective effect was investigated in infants infected before blood collection based on the assumption that infants without malaria infection were unable to develop their own Abs.

For the analyses regarding the protection of antibodies against malaria infection, 28 hypotheses were tested, corresponding to the 28 Ab responses studied. Results were not corrected for multiple testing as this study was considered as explanatory, designed to generate new hypotheses [33]. Furthermore, applying Bonferroni or Benjamini–Hochberg corrections would be over conservative, leading to a significant reduction in the study's ability to assess true differences. Indeed, computations of these corrections assume that the multiple tests are independent. However, Ab concentrations of the different antigens are correlated, as are the Ab concentrations of IgG subclasses (mainly IgG1 and IgG3). The results highlighted were those exhibiting multiple lines of evidence (among the Ab responses or among the outcomes studied).

## Results

### Characteristic of the study population

The Additional file 2: Table S1 shows the major features of the Beninese mothers and their infants, emphasizing that: (i) most women (n = 300; 76%) were followed-up at the Sekou health centre, (ii) the majority of women (n = 277; 70%) were from Aizo ethnic group, (iii) 62 (15%) were primigravidae, (iv) 146 (24%) of the women were evaluated at the ANV2 during the rainy season, (v) 42 (11%) presented placental infection detected at delivery, (vi) the majority of the infants (n = 290; 95%) used mosquito nets, (vii) 35 newborns (9%) exhibited low birth weight, and (viii) compared to the first year, more malaria episodes were detected at the second-year of follow-up. For women, prevalence of peripheral malaria infection was the highest at inclusion, at ANV1 (16%) and the lowest at ANV2 (4%). For infants, the prevalence was 13% at 12 months and 17% at 24 months. Prevalence of malaria infections at visits and the number of individuals with at least one malaria episodes between the visits are described in Additional file 2: Table S1.

### Antibody concentrations dynamics against P. falciparum asexual stage antigens

The measurements of Ab responses were obtained for 375 women and for 287 infants. This number varied according to the visits and antigens, mainly as a result of an insufficient quantity of plasma collected. The number of Ab responses quantified at each time point is described in Additional file 2: Table S1.

**Fig. 1:**
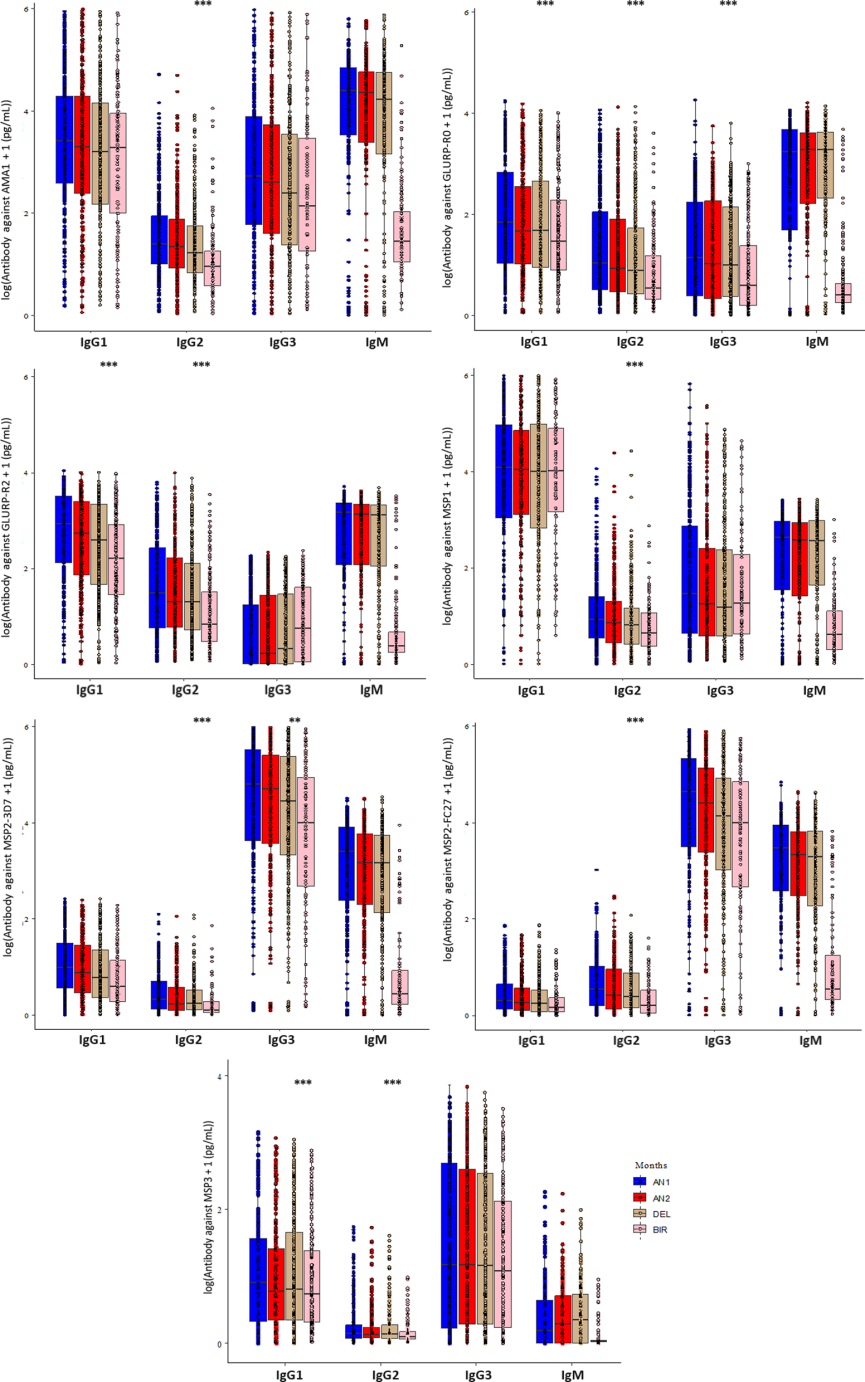
Antibody kinetics during pregnancy and at birth. Overview of the specific asexual stage antigens IgG1, IgG2, IgG3, and IgM Ab concentrations (pg/mL, median and range) against the AMA1, GLURP-R0, GLURP-R2, MSP1, MSP2-3D7, MSP2-FC27 and MSP3 antigens in mothers at the antenatal visits (ANV1 and ANV2), at delivery (DEL) and at birth (BIR, cord blood). The Significant *p*-value is (*p* < 0.05). Nominal *p*-value levels are indicated by: *p* < 0.1; *p < 0.05; ** *p* < 0.01; *** *p* < 0.001. Colored dots in each column indicate individual data points and their distribution. Only differences in IgGs levels between mother at delivery (DEL) and newborn at birth (BIR) analyzed by paired t-test are shown in the figure. IgM levels difference between delivery (DEL) and birth (BIR) are not shown, as it is known that there is no transfer of IgM from mother to newborn. Using Tukey's pairwise method, the differences in other antibody levels at each time point with reference to the start point are summarized in Additional file 2: Table S2

Anti-asexual stage Ab concentrations were analysed at several time settings, including: i) from ANV1 (before the first IPTp) to delivery (DEL), ANV1 to ANV2, and ANV2 to DEL, to assess Ab concentration evolution during gestation in a group of women who are receiving IPTp, ii) at delivery in the peripheral and cord blood, to evaluate the Ab transfer from mother to child, and iii) in infants from birth (cord blood) till 24 months of life, to evaluate the dynamics of Ab transferred and the Ab acquisition during infancy. Figure 1 summarizes all these results.

#### Anti-asexual stage antibody concentrations in mothers during pregnancy

Overall, the concentrations of anti-asexual stage Abs were higher at ANV1 when compared to delivery. For all IgG anti-asexual stage subclasses, a significant decrease of the responses was observed, except for those specific to MSP3, for IgG1 against GLURP-R0, and IgG3 against GLURP-R2 (Additional file 2: Table S2 and Fig. 1). For most of Ab responses, the results show that anti-asexual stage Ab concentration progressively decreased along gestation with an intermediate concentration at ANV2. This decrease was more important between ANV1 and ANV2 after the first dose of IPTp than between ANV2 and delivery (Additional file 2: Table S2 and Fig. S1). This early decrease is particularly observed for IgG2, which showed a significant decrease between ANV1 and ANV2 for all antigens (Additional file 2: Table S2 and Additional file 2: Fig. S1). For all IgM anti-asexual stage antigens, no significant differences were observed for all comparisons. All results concerning the comparison of anti-asexual stage Abs responses between ANV1, ANV2 and DEL are presented in detail in Additional file 2: Table S2. Significant differences in IgGs between maternal and cord blood levels at delivery were mentioned in Fig. 1.

#### Anti-asexual stage antibodies in mother and newborn

To understand the relationship between the anti-asexual stage IgG concentration of the mother at delivery and the newborn, the IgG Ab concentration of mother in plasma was compared with the infant IgG Ab concentration in cord blood.

The comparisons between the specific asexual stage IgG Abs in mothers and newborns revealed no significant differences, except for IgG2 against AMA1, IgG1/2/3 against GLURP-R0, IgG1/2 against GLURP-R2, IgG2 against MSP1, IgG2/3 against MSP2-3D7, IgG2 against MSP2-FC27, and IgG1/2 against MSP3, which were decreased in the cord blood (Fig. 1).

Strong and very strong correlations were observed principally for IgG1 and IgG3 concentrations between maternal and cord blood levels at delivery; whereas IgG2 exhibited high correlation (r > 0.60) (Additional file 2: Fig. S2). Since IgM does not cross the placenta, there was no correlation between Ab concentrations of all tested anti-asexual stage Abs in the cord blood and mother’s blood at delivery.

#### Acquisition of anti-asexual stage antibodies during infancy

**Fig. 2:**
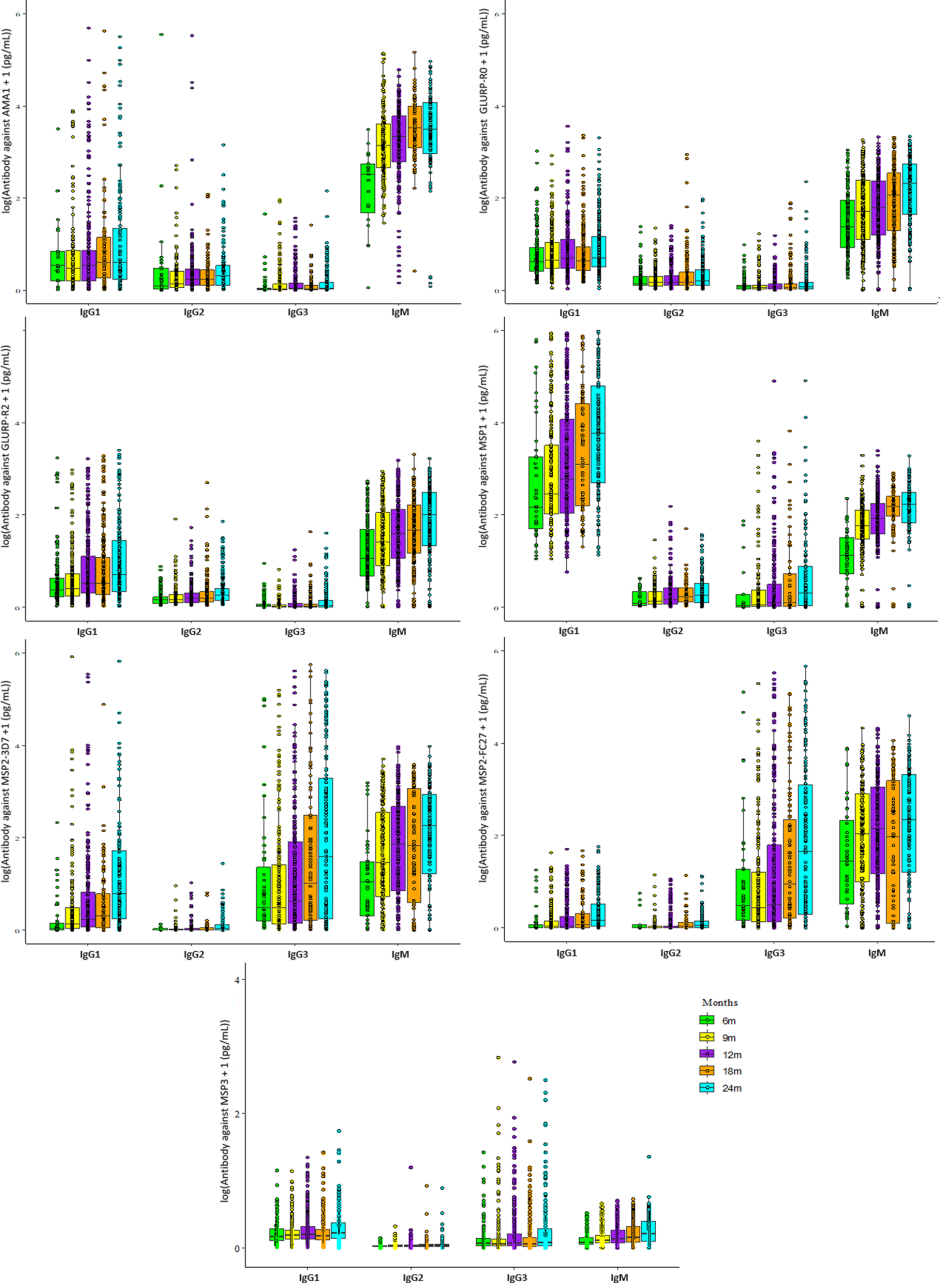
Antibody kinetics during infancy. Overview of the specific asexual stage antigens IgG1, IgG2, IgG3, and IgM Ab concentrations (pg/mL, median and range) against the AMA1, GLURP-R0, GLURP-R2, MSP1, MSP2-3D7, MSP2-FC27 and MSP3 antigens in infants from birth (BIR, cord blood) until the age of two years (at 6, 9, 12, 18, and 24 months). Colored dots in each column indicate individual data points and their distribution. Using Tukey's pairwise method, the differences in other antibody levels at each time point with reference to the start point are summarized in Additional file 2: Table S2

In infancy, the lower concentrations of the specific asexual stage IgG Abs primarily occurred at the age of six months, putatively coinciding with the loss of maternal IgG Abs. Thereafter, the concentrations of almost all anti-asexual stage Abs increased along the 24 months of follow-up with predominant responses for IgM. In the majority of the comparisons of Ab concentrations acquired at 9th, 12th, 18th and 24th months with the Ab concentration developed at the age of 6 months, a significant acquisition of Ab concentration appeared at the age of 12 months (Fig. 2 and Additional file 2: Table S2).

### Antibody IgG transfer from mother to newborn

#### Ability of maternal IgG to cross the placenta

Significant differences between IgG subclasses were observed when the ratios of specific Abs transferred from mother to newborn were compared (Additional file 2: Table S3). Ratio values were higher for IgG1 and IgG3 compared to IgG2 (Additional file 2: Table S3). Moreover, IgG1 appeared to be the subclass exhibiting the highest transfer ratio. When compared to IgG3, IgG1 transfer was significantly higher among four (GLURP-R0, GLURP-R2, MSP1 and MSP3) to seven responses (Additional file 2: Table S3).

Differences in IgG transfer were also observed between vaccine candidate antigens; however, a specific Ab response, which was better transferred than the others, cannot be identified. For IgG1 and IgG2 responses, the anti-MSP1 and anti-AMA1 Abs were the most transferred from the mother to the newborn, respectively (Additional file 2: Table S4). The Ab transfer of these two responses was significantly higher than those of GLURP-R0 and GLURP-R2 (Additional file 2: Table S4). For IgG3 response, anti-AMA1 Abs had the higher value of Ab transfer (ratio = 1) and appeared to be the best transferred of all IgG Abs. However, no significant difference in terms of Ab transfer was observed compared to the other Ab responses (Additional file 2: Table S4).

#### Evaluation of the factors that influence IgG transfer from mother to infant

Ab levels at ANV2 and delivery, malaria infection during pregnancy, ethnic group and the Ballard score appeared to impact on the transfer of Abs from mother to newborn. At delivery, the levels of maternal Abs were negatively associated with the transfer of all IgG Abs responses; except for the IgG3 against MSP2-3D7 (p = 0.06), AMA1 (p = 0.43), and IgG2 against MSP1 (p = 0.91) (Table 1 and Additional file 2: Table S5). In contrast, the levels of maternal Abs during pregnancy (ANV2) were associated with a better transfer of Abs, mainly with the maternal cytophilic (IgG1 and IgG3) subclasses (Table 1). Occurrence of malaria infection during pregnancy was associated with a lower IgG transfer to the newborn. This observation encompasses the majority of IgG1 against the candidate vaccine antigens, except for anti-MSP2 (Table 1). For IgG2 and IgG3 responses, an association with malaria infection during pregnancy is observed only for IgG2 against MSP1 and IgG3 against GLURP-R0 (Table 1 and Additional file 2: Table S5). Placental infection appeared to be associated with a lower level of IgG3 transfer for IgG3 against MSP1 and MSP2-3D7 (Table 1). No collinearity was observed between the variables for all the models (VIF < 5).

**Table 1:** Factors influencing IgG1 and IgG3 transplacental transfer, emphasizing multivariate linear regressions for the asexual stage antigens

Variables	*Estimate [95%CI]	*p*-value	*Estimate [95%CI]	*p*-value
	IgG1 to AMA1 transfer		IgG3 to AMA1 transfer	
Maternal specific Ab at delivery	−0.167 [−0.26; −0.074]	**0.001**	NR	NR
^1^Malaria during pregnancy	−0.129 [−0.264; 0.005]	0.06	NR	NR
^1^Placental malaria	NI	NI	NI	NI
Maternal specific Ab at ANV2	0.149 [0.064; 0.233]	**0.001**	NR	NR
Ballard score	NR	NR	NR	NR
Ethnic groups: Tori			Reference	
Other groups			NR	NR
Fon			NR	NR
	IgG1 to GLURP−R0 transfer		IgG3 to GLURP−R0 transfer	
Maternal specific Ab at delivery	−0.137 [−0.19; −0.084]	** < 0.001**	−0.109 [−0.155; −0.064]	** < 0.001**
^1^Malaria during pregnancy	−0.14 [−0.25; −0.031]	**0.012**	−0.104 [−0.202; −0.006]	**0.038**
^1^Placental malaria	NI	NI	NI	NI
Maternal specific Ab at ANV2	0.064 [0.015; 0.113]	**0.011**	0.069 [0.027; 0.112]	**0.002**
Ballard score	0.013 [−0.005; 0.032]	0.163	NR	NR
Ethnic groups: Tori			Reference	
Other groups			−0.131 [−0.281; 0.019]	0.086
Fon			NR	NR
	IgG1 to GLURP−R2 transfer		IgG3 to GLURP−R2 transfer	
Maternal specific Ab at delivery	−0.062 [−0.116; −0.008]	**0.023**	−0.058 [−0.076; −0.04]	** < 0.001**
^1^Malaria during pregnancy	−0.11 [−0.216; −0.003]	**0.043**	NR	NR
^1^Placental malaria	NI	NI	NI	NI
Maternal specific Ab at ANV2	0.046 [−0.001; 0.09]	**0.05**	NR	NR
Ballard score	NR	NR	NR	NR
Ethnic groups: Tori			Reference	
Other groups			NR	NR
Fon			0.126 [0.004; 0.247]	**0.043**
	IgG1 to MSP1 transfer		IgG3 to MSP1 transfer	
Maternal specific Ab at delivery	−0.061 [−0.112; −0.01]	**0.019**	−0.094 [−0.163; −0.025]	**0.008**
^1^Malaria during pregnancy	−0.202 [−0.343; −0.061]	**0.005**	NI	NI
^1^Placental malaria	NI	NI	−0.267[−0.44; −0.09]	**0.002**
Maternal specific Ab at ANV2	0.052 [0.004; 0.099]	**0.033**	0.056 [−0.01; 0.122]	0.097
Ballard score	0.018 [−0.004; 0.04]	0.1	0.025 [0.007; 0.044]	**0.009**
Ethnic groups: Tori			Reference	
Other groups			NR	NR
Fon			NR	NR
	IgG1 to MSP2−3D7 transfer		IgG3 to MSP2−3D7 transfer	
Maternal specific Ab at delivery	−0.076 [−0.133; −0.019]	**0.01**	−0.068 [−0.141; 0.004]	0.064
^1^Malaria during pregnancy	−0.139 [−0.314; 0.036]	0.11	NI	NI
^1^Placental malaria	NI	NI	−0.254 [−0.486; [−0.021]	**0.032**
Maternal specific Ab at ANV2	NR	NR	0.06 [−0.003; 0.123]	0.064
Ballard score	0.03 [0.003; 0.057]	**0.031**	0.045 [0.022; 0.069]	** < 0.001**
Ethnic groups: Tori			Reference	
Other groups			NR	NR
Fon			0.266 [0.06; 0.473]	**0.012**
	IgG1 to MSP2−FC27 transfer		IgG3 to MSP2−FC27 transfer	
Maternal specific Ab at delivery	−0.129 [−0.188; −0.07]	** < 0.001**	−0.119 [−0.181; −0.057]	** < 0.001**
^1^Malaria during pregnancy	NR	NR	NR	NR
^1^Placental malaria	NI	NI	NI	NI
Maternal specific Ab at ANV2	0.078 [0.025; 0.131]	**0.004**	0.075 [0.016; 0.134]	**0.013**
Ballard score	NR	NR	0.041 [0.015; 0.066]	**0.002**
Ethnic groups: Tori			Reference	
Other groups			NR	NR
Fon			0.304 [0.09; 0.517]	**0.006**
	IgG1 to MSP3 transfer		IgG3 to MSP3 transfer	
Maternal specific Ab at delivery	−0.102 [−0.136; −0.07]	** < 0.001**	−0.04 [−0.068; −0.012]	**0.005**
^1^Malaria during pregnancy	−0.143 [−0.257;—0.029]	**0.01**	−0.083 [−0.198; 0.033]	0.16
^1^Placental malaria	NI	NI	NI	NI
Maternal specific Ab at ANV2	NR	NR	NR	NR
Ballard score	0.017 [−0.001; 0.035]	0.06	NR	NR
Ethnic groups: Tori			Reference	
Other groups			NR	NR
Fon			0.108 [−0.04; 0.256]	0.15

^*^ refers to the coefficient of linear regression; i.e., the negative or positive effect of the variables on the trans−placental Ab transfer and the 95% confidence interval. ^1^ Only one of the following variables, placental malaria or *P. falciparum* infection during pregnancy was included in the final multivariate model because both are correlated; the associations presenting the lowest *p*-values were selected. Significant co-variates and *p*-values (*p* < 0.05) are shown in bold. Levels of nominal *p*-values are indicated by: •*p* < 0.1; *p < 0.05; ** *p* < 0.01; ***p < 0.001. NI: variable Not Included in the final model. NR: not relevant for co-variables with a *p*-value greater than 0.20 in the final multivariate model

### Protection of maternal antibodies against malaria infection during pregnancy

#### Antibody protection against peripheral infections

**Fig. 3:**
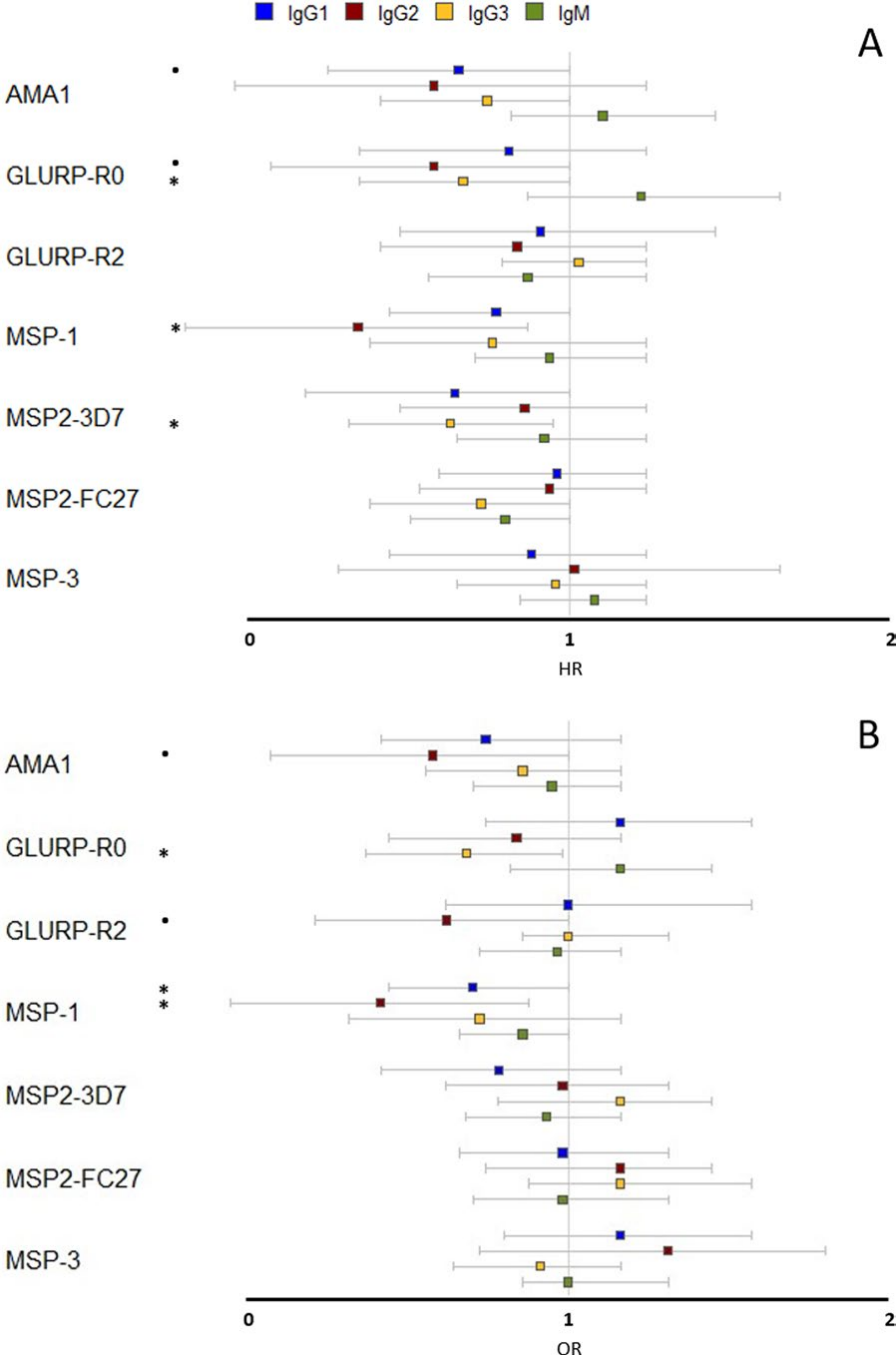
**A** Maternal antibodies protection against peripheral malaria infections during pregnancy. Hazard ratios (HR) and 95% confidence intervals obtained from a Cox regression model are represented for each Ab responses measured at ANV2. A hazard ratio < 1 indicates that Ab response is associated with a decrease risk of malaria infection; whereas a hazard ratio > 1 indicates that Ab response is associated with a higher risk of malaria infection. Analyses were adjusted on the transmission season at the time of ANV2 (dry versus rainy season), malaria infection before ANV2, gravidity status (Primigravidity versus Multigravidity) and the possession of TV. **B** Maternal antibodies protection against placental malaria infection. Odds ratios (OR) and 95% confidence are represented for each Ab responses measured at ANV2. An odd ratio < 1 indicates that Ab response is associated with a decrease risk of placental malaria infection at delivery. Results were adjusted on the ethnic groups and age group of the mothers. For both, significant* p*-values are (*p* < 0.05) and levels of nominal *p*-values are indicated by: •*p* < 0.1; *p < 0.05; ** *p* < 0.01; ***p < 0.001

The association between Abs directed against candidate vaccine antigens and the onset of the first malaria infection was investigated during pregnancy from the ANV2 to delivery. The result of the analysis by Cox regression revealed that certain IgG Ab responses appeared to be associated with a protection against peripheral infection. The responses of IgG3 directed against GLURP-R0 (*p* = 0.04) and MSP2-3D7 (*p* = 0.02), IgG2 against MSP1 (*p* = 0.01) are associated with reduction of *P. falciparum* infection risk at a nominal p-value < 0.05 (Fig. 3A).

#### Antibody protection against placental infection

The logistic regression regarding the impact of maternal Ab concentration at ANV2 on the placental infection showed that IgG1 (*p* = 0.04) and IgG2 (*p* = 0.01) responses directed against MSP1, and IgG3 directed against GLURP-R0 (*p* = 0.03) are associated with a protection against placental malaria (Fig. 3B) with a nominal *p*-value < 0.05.

It can be noted that IgG2 response against MSP1 and IgG3 against GLURP-R0 were found to be associated with both a lower risk of re-infection during pregnancy and a lower risk of placental malaria.

### Association of maternal antibodies with malaria protection during infancy

The protective effect of maternal Abs during the first months of life was analysed using the time to the onset of the first malaria infection and the number of malaria attacks over the entire follow-up. The results showed no evidence of maternal Ab protection (Additional file 2: Table S6).

### Association between antibody responses and malaria protection in infants from one to two years of life

The effect of the Ab responses against candidate vaccine antigens was studied in infants between the ages of 12 to 18 months and 18 to 24 months. A Cox model was used to assess the effect of Abs on the occurrence of symptomatic malaria infections. The results revealed that there is no protection conferred by Abs at 12 months of age (Additional file 2: Table S7). Between 18 and 24 months of age, anti-MSP3 IgM is associated with a protection against the onset of symptomatic malaria in the infants of the subgroup 1. In the subgroup 2, IgM Abs against MSP2-FC27 and MSP3 are associated with malaria protection, while in the subgroup 3, all IgM Abs against MSP1, MSP2 and MSP3 are associated with malaria protection (Table 2). Analysis of the results in subgroup 1, 2 and 3 showed that Ab responses were associated with a decreased risk of symptomatic malaria infection in case of recent *P. falciparum* infection (in the last 3 months). In subgroup 3, after adjustment for multiple testing using the Benjamin-Hocheberg correction, which is a stringent correction given the correlations existing between the Ab reponses in our study, the association for IgM to MSP2-FC27 remains significant (adjusted p-value: 0.002); a tendency is observed for IgM to MSP1 and IgG3 to MSP1 (adjusted *p* = 0.08 and 0.09, respectively). It is worth noting that IgG3 antibody levels against MSP1 in the three subgroups were associated with an increase with the onset of symptomatic malaria infections.

**Table 2:** Hazard ratios and 95% confidence intervals of Ab responses against MSP family between 18–24 months

	**Subgroup 1**	**Subgroup 2**	**Subgroup 3**
IgG1 to MSP1	0.97 [0.78; 1.20]	1.03 [0.80; 1.32]	0.96 [0.75; 1.24]
IgG2 to MSP1	1.25 [0.86; 1.82]	1.29 [0.83; 1.99]	1.20 [0.77; 1.87]
IgG3 to MSP1	1.31 [1.04; 1.63]^*^	1.40 [1.10; 1.76]^**^	1.35 [1.07; 1.69]^*^
IgM to MSP1	1.02 [0.85; 1.23]	0.95 [0.76; 1.18]	0.74 [0.60; 0.92]^**^
IgG1 to MSP2-3D7	1.07 [0.93; 1.24]	**1.15 [0.99; 1.34]**^**•**^	1.13 [0.95; 1.34]
IgG2 to MSP2-3D7	0.93 [0.74; 1.17]	0.99 [0.77; 1.27]	0.98 [0.74; 1.29]
IgG3 to MSP2-3D7	0.99 [0.86; 1.15]	1.04 [0.86; 1.25]	1.06 [0.87; 1.27]
IgM to MSP2-3D7	0.94 [0.81; 1.09]	0.88 [0.75; 1.03]	0.84 [0.71; 0.99]^*^
IgG1 to MSP2-FC27	0.97 [0.85; 1.10]	0.99 [0.86; 1.15]	0.96 [0.78; 1.17]
IgG2 to MSP2-FC27	1.13 [0.89; 1.41]	1.17 [0.93; 1.47]	1.12 [0.89; 1.40]
IgG3 to MSP2-FC27	1.04 [0.92; 1.18]	1.05 [0.90; 1.22]	1.07 [0.89; 1.27]
IgM to MSP2-FC27	0.92 [0.82; 1.03]	0.87 [0.77; 0.99]^*^	0.80 [0.71; 0.90]^***^
IgG1 to MSP3	0.96 [0.77; 1.18]	0.94 [0.73; 1.22]	0.95 [0.74; 1.23]
IgG2 to MSP3	1.18 [0.88; 1.58]	1.12 [0.81; 1.55]	1.16 [0.86; 1.56]
IgG3 to MSP3	1.07 [0.96; 1.20]	**1.14 [0.99; 1.30]**^**•**^	**1.13 [0.99; 1.30]**^**•**^
IgM to MSP3	0.87 [0.77; 0.98]^*^	0.86 [0.77; 0.97]^*^	0.88 [0.78; 0.99]^*^

Only Ab responses with a significant effect on the risk of symptomatic malaria infection are presented. Hazard ratios were obtained from a multivariate Cox model in the three infant’s subgroups: 1) children (n = 155) with a least two malaria infections throughout the follow-up (exposed children), 2) exposed children (n = 133) with at least one infection, and 3) exposed children (n = 106) with at least one infection in the last three months. A hazard ratio < 1 indicates that Ab concentration is associated with a decreased risk of malaria infection; whereas a hazard ratio > 1 indicates that Ab concentration is associated with a higher risk of malaria infection. Analyses were adjusted for environmental risk, transmission season, low birth weight and marital status. Significant co-variates and *p*-values (*p* < 0.05) are shown in bold. Levels of nominal *p*-values are indicated by: •*p* < 0.1; *p < 0.05; ** *p* < 0.01; ***p < 0.001

## Discussion

Identification of the best candidate vaccine antigens in vulnerable populations and under natural condition of malaria exposure is crucial to design the vaccines including antigens derived from different stages of *P. falciparum* life cycle. The results of the present study clearly showed that antibody responses related to malaria protection differed during pregnancy and infancy. IgG2 against MSP1 and IgG3 against GLURP-R0 tend to be associated with protection against malaria during pregnancy while IgM against MSP1, MSP2-3D7, MSP2-FC27, and MSP3 antigens appear to protect infants from 18 to 24 months of age. The robustness of this study relies on the availability of epidemiological, entomological, and immunological data actively collected during a four-year period from 400 mothers and their infant, evaluating 28 Ab responses specific to seven malaria candidate vaccine antigens. Entomological data, together with environmental and climatic data allowed modeling an individual risk of environmental exposure [26], which were used in this study to adjust the analyses between the Ab concentrations and the risk of malaria infection in infants.

Maternal Abs against asexual stage antigens gradually decreased along pregnancy, while in infants a progressive decrease of maternal-acquired Abs was observed along the first six months of life. Thereafter, the acquired anti-malarial Abs progressively increased in infants until the age of 24 months. When the levels of Abs from ANV1 to ANV2 and from ANV2 to delivery were compared, a decrease in almost all IgG responses was observed. As described in other works, this decrease of antimalarial Abs was associated with the IPTp intake. The differences of Ab response was more important after the intake of two IPTp doses. Indeed, several previous studies have shown that the decreased Ab concentration observed during pregnancy was associated with IPTp [33, 34]. Another study also reported decreased levels of IgG against GLURP from the baseline to IPTp1 and from IPTp2 to IPTp3 [33]. The hypothesis to explain the effect of IPTp drug on anti-malarial Ab could be the decrease of the immune system stimulation due to the reduced exposure or parasite clearance after IPTp intake [33]. However, other factors may influence the Ab decline, such as the use of ITN [35, 36] or the phenomenon of immune tolerance [37]. Although the current study did not collect information on ITNs before the first visit (ANV1), it is possible that ITNs have contributed to the reduction of mosquito-vector contact decreasing the Ab levels. Considering that pregnant women tolerate and do not reject their semi allogenic fetus, it is possible that the expression of immunoregulatory molecules, such as HLA-G [38], may account for the decreased levels of specific Abs, demanding further studies to clarify this issue. Interestingly, the anti-malarial IgG2 concentrations decreased more rapidly than those of IgG1 and IgG3 after the first dose of IPTp (ANV2); however, there was no clear explanation for this observation.

Regarding the Ab transfer from the mother to the newborn, the results of this study showed closely similar IgG concentration at delivery in maternal blood and in the newborn's cord blood, supporting the placental transfer of IgG [39, 40]. IgG1 and IgG3 Abs were highly transferred from the mother to newborn, as previously reported [41, 42]. Moreover, specific Abs against AMA1 and MSP1 antigens were highly transferred from the mother to the newborn. This observation could be explained by the high immunogenicity of these antigens [44]. Interestingly, peripheral *P. falciparum* infection during pregnancy was associated with a reduced placental transfer of Abs, as observed in this study. Previous studies reported that high IgG levels, peripheral infections in pregnancy or placental malaria were associated with the low transplacental Ab transfer [14, 45–47]. Indeed, maternal hypergammaglobulinaemia [14, 45, 48], particularly high IgG cord blood levels may negatively impact on the transplacental transfer of IgG against *P. falciparum* antigens [44, 49, 50], suggesting a saturation of FcRn receptors,. Early gestational age and primigravida women [46], and the presence of other infections such as HIV infections [51, 52; 48] have also been associated with decreased Ab placental transfer. Further research is required to elucidate the mechanisms by which maternal peripheral *P. falciparum* infection reduce transplacental Ab passage. Noteworthy, as shown in this study, the majority of infections were observed between ANV2 (after the second dose of IPTp) and delivery, supporting the hypothesis of a FcRn saturation at the end of pregnancy. Additionally, it was also found that high Ab levels at delivery reduced the placental transfer. All these findings confirm the observations supported by previous studies, which reported that the placental transfer is lower in areas of high malaria endemicity, where the maternal Ab concentration is high compared to areas of low transmission [41, 46].

An expected acquisition of anti-asexual stage Ab was observed during the first two years of life, as previously reported by other studies [32, 47–51]. The rise in anti-malarial Ab concentrations from 6 to 24 months reveals a progressive immunity acquisition mainly due to repeated exposure to *P. falciparum* during the first 24 months of life.

Overall, this study of the humoral protection against malaria along pregnancy showed that IgGs appear to be more effective than IgM. Indeed, IgG1 and IgG2 anti-MSP1, IgG2 anti-GLURP-R0, and IgG3 anti-MSP2-3D7 were associated with a protection against malaria infection and/or placental malaria during pregnancy. Although these associations do not remain significant after the Bonferroni correction for multiple testing, all effects are suggestive of protection. Similar studies have also reported a protective role of IgGs against *P. falciparum* malaria during pregnancy [52–55]; however, other studies have not [56–58]. This discrepancy may be explained by the heterogeneity of malaria exposure, distinct specificities of Ab against particular antigens, differential clinical monitoring of patients, and distinct methods for *P. falciparum* detection. Although the majority of vaccine studies against *P. falciparum* antigens during pregnancy have focused on the antigen variant VAR2CSA of the *P. falciparum* erythrocyte membrane protein 1 (PfEMP1) family that can bind to its receptor on placental syncytiotrophoblasts [59–62], it was shown that Ab responses against non-pregnancy specific *P. falciparum* antigens may also play an important role on the protection against gestational malaria. Furthermore, an unexpected association between anti-asexual stage IgG2 and malaria protection during pregnancy was observed here as well as in other malaria studies [63, 64, 72], deserving additional studies directed towards this specific Ab.

Regarding the protection conferred by Ab against *P. falciparum* asexual stage antigens along infancy, a protective effect of the maternal Abs until 12 months of age was not observed. The lack of evidence of a protective maternal Abs in infancy can be explained by the use of ITNs [73, 74] and fetal hemoglobin [75, 76]. Although no protection was observed along the first 12 months of follow-up, higher concentration of IgM against MSP1, MSP2-3D7, MSP2-FC27 and MSP3 were associated with lower risk of malaria infection between infants aged 18 to 24 months. Interestingly, the majority of acquired immunity against *P. falciparum* antigens in infants has focused on IgGs [15, 32, 58], whereas IgM has been associated with malaria protection in children over the age of five years [18].

To our knowledge, this is the first study to report an association between protective against malaria and anti-asexual stage specific IgM in the first years of life. Two studies using MSP1, MSP2 and AMA1 antigens have reported associations between the functional activity of IgM directed to asexual stage antigens and protection against malaria [18, 77]. The first study showed a reduction in clinical malaria risk with asexual stage specific IgM, which persisted up to 12 months in children aged 5–14 years [18], while the second one did not show low susceptibility with asexual stage specific IgM in older individuals (3 months to 36 years) [77]. Several lines of evidence indicate better performance of IgM anti-malarial response when compared to IgG, including; i) IgM inhibited *P. falciparum* parasitaemia better than IgG by complement-mediated lysis [18] or similarly to IgG by direct neutralization and opsonic phagocytosis [77], ii) somatically hypermutated memory B cells present rapid induction and high affinity IgM response to secondary malaria infection [18, 78, 79], as well as IgG whose affinity increases over time [80], iii) the study of malaria susceptibility in Malian sympatric groups reveals that the Fulani ethnic group is resistant to malaria infection when compared to the Dogon group, and *P. falciparum*-specific IgM response is primarily observed in the former group [81–83]. Taken together, these observations indicate that asexual stage specific IgG Abs have to pass through proliferation, differentiation and affinity maturation to achieve a protective activity, gradually disrupting and replacing the protective effect of IgM with those of IgGs, which are the major protective anti-*P. falciparum* antibodies, as observed in this cohort of pregnant women. The understanding of kinetics of IgM and IgG anti-*P. falciparum* Abs along infanthood may disclose the maturity processes of these Abs, providing the tools to design effective vaccines to control malaria infection in this age group.

## Conclusion

In conclusion, IgG2 against MSP1 and IgG3 against GLURP-R0 were associated with malaria protection during pregnancy, while IgM against MSP1, MSP2 and MSP3 were associated with malaria protection during infancy, suggesting a possible delay on the transition from IgM to IgG anti-*P. falciparum* Ab in infancy. Functional studies on the biological activity of anti-asexual stage Abs are crucial to confirm their involvement in malaria protection, shedding light into the development of promising malaria candidate vaccine antigens. The present study supports the evidence that IgM during infanthood plays an effective role in the acquisition of anti-malarial immunity; the next challenge is the understanding of the biological mechanisms associated with the development of these specific anti-*P. falciparum* asexual stage Abs.

## Ethics approval

The institutional review boards of the Comité Consultatif de Déontologie et d'Éthique from the Institut de Recherche pour le Développement (France), the Ethic Committee of the Faculty of Health Sciences (University of Abomey-Calavi, Benin) and the Human Research Ethics Committee of the University Hospital of the Ribeirão Preto Medical School of the University of São Paulo (Brazil) approved all study protocols included in this paper. Before inclusion, the study was explained and written informed consent of women was obtained in the presence of a witness, with thumbprints provided if women could not read and/or write.

## Conflicts of interest

The authors declare no conflict of interest.

### Supplementary Information

Additional file1 (DOCX 27 KB).

Additional file2 (RTF 264451 KB).

## Data Availability

The datasets used for analyses during this study are available from the Corresponding author on request.
