# Supplementary material for: IgG and IgM responses to the Plasmodium falciparum asexual stage antigens reflect respectively protection against malaria during pregnancy and infanthood
Source: Malar J. 2024 May 19;23:154. doi: 10.1186/s12936-024-04970-7 (PMC11103834; doi:10.1186/s12936-024-04970-7)
Supplement: Supplementary file 1 — Additional file1 (DOCX 27 KB). [file 12936_2024_4970_MOESM1_ESM.docx]

**Additional materials**

**Details on the analyses of the protection of antibodies against malaria infection**

**Covariates considered**

- In analyses of the protection of antibodies against peripheral infections between ANV2 and delivery : the age of the mother (in four 5-year groups), gravidity (primigravidae *versus* multigravidae), schooling of the mother, ethnic group, socioeconomic data as described in the mother-child follow-up, IPTp group, the transmission season at ANV2 and the presence of an infection before ANV2.

- In analyses of the protection of antibodies against placental malaria : the age of the mother (in four 5-year groups), gravidity (primigravidae *versus* multigravidae), schooling of the mother, ethnic group, socioeconomic data as described in the mother-child follow-up, IPTp group and the transmission season at ANV2.

- In analyses of the protection of antibodies in infants: the age of the mother (in four 5-year groups), gravidity (primigravidae *versus* multigravidae), schooling of the mother, ethnic group, socioeconomic data, anemia at delivery (<11 g/dL), placental malaria, the presence of a malaria infection during pregnancy and the risk of environmental exposure.

**Analysis strategy**

Whatever the outcome considered (time to re-infections or placental malaria in mothers; the number of malaria attacks or time to infections in infants) the same strategy was applied. Each covariate was first tested in an univariate analysis. Then Ab responses were tested one by one in a multivariate model including the covariates associated with a *p*<0.20 in univariate analyses.

**Adjustment on the risk of environmental exposure**

Valmaseda and collaborators emphasized the importance of adjusting analyses on malaria exposure to assess the relationship between the Abs and the protection against malaria. The heterogeneity of malaria exposure may be due to several factors such as local transmission intensity, differential mosquito attraction as well as protection measures set up by the mothers. In this study, analyses of antibody protection in infants were adjusted for environmental exposure risk, estimated once a month for each infant as described in the method section “Prediction of environmental risk by entomological and geographic variables”.

In the analyses on time to infection (with a Cox model), this variable was considered as a time-dependent covariate. The multivariate analyses were also adjusted for the transmission season (rainy season of 2010, 2011 and 2012), the intensity of which can vary from one year to the next.

In the analyses of the number of malaria attacks (with a negative binomial model), the multivariate analyses were adjusted to the average of the environmental risk estimates for the infant over one year.
