# Supplementary material for: IgG and IgM responses to the Plasmodium falciparum asexual stage antigens reflect respectively protection against malaria during pregnancy and infanthood
Source: Malar J. 2024 May 19;23:154. doi: 10.1186/s12936-024-04970-7 (PMC11103834; doi:10.1186/s12936-024-04970-7)
Supplement: Supplementary file 2 — Additional file2 (RTF 264451 KB). [file 12936_2024_4970_MOESM2_ESM.rtf]

Supporting Information
Figures
Figure 1. Antibody kinetics during pregnancy and at birth
Overview of the specific asexual stage antigens IgG1, IgG2, IgG3, and IgM Ab concentrations (pg/mL, median and range) against the AMA1, GLURP-R0, GLURP-R2, MSP1, MSP2-3D7, MSP2-FC27 and MSP3 antigens in mothers at the antenatal visits (ANV1 and ANV2), at delivery (DEL) and at birth (BIR, cord blood). The Significant p-value is (p < 0.05). Nominal p-value levels are indicated by: p < 0.1; *p < 0.05; ** p < 0.01; *** p < 0.001. Colored dots in each column indicate individual data points and their distribution. Only differences in IgGs levels between mother at delivery (DEL) and newborn at birth (BIR) analyzed by paired t-test are shown in the figure. IgM levels difference between delivery (DEL) and birth (BIR) are not shown, as it is known that there is no transfer of IgM from mother to newborn. Using Tukey's pairwise method, the differences in other antibody levels at each time point with reference to the start point are summarized in Supplementary Table 2. 


Figure 2. Antibody kinetics during infancy
Overview of the specific asexual stage antigens IgG1, IgG2, IgG3, and IgM Ab concentrations (pg/mL, median and range) against the AMA1, GLURP-R0, GLURP-R2, MSP1, MSP2-3D7, MSP2-FC27 and MSP3 antigens in infants from birth (BIR, cord blood) until the age of two years (at 6, 9, 12, 18, and 24 months). Colored dots in each column indicate individual data points and their distribution. Using Tukey's pairwise method, the differences in other antibody levels at each time point with reference to the start point are summarized in Supplementary Table 2. 


Figure 3. A.) Maternal antibodies protection against peripheral malaria infections during pregnancy. Hazard ratios (HR) and 95% confidence intervals obtained from a Cox regression model are represented for each Ab responses measured at ANV2. A hazard ratio<1 indicates that Ab response is associated with a decrease risk of malaria infection; whereas a hazard ratio>1 indicates that Ab response is associated with a higher risk of malaria infection. Analyses were adjusted on the transmission season at the time of ANV2 (dry versus rainy season), malaria infection before ANV2, gravidity status (Primigravidity versus Multigravidity) and the possession of TV. B.) Maternal antibodies protection against placental malaria infection. Odds ratios (OR) and 95% confidence are represented for each Ab responses measured at ANV2. An odd ratio<1 indicates that Ab response is associated with a decrease risk of placental malaria infection at delivery. Results were adjusted on the ethnic groups and age group of the mothers. For both, significant p-values are (p < 0.05) and levels of nominal p-values are indicated by: p < 0.1; *p < 0.05; ** p < 0.01; ***p< 0.001. 

Supplementary Figure 1. Effect of the IPTp drugs on the modulation of anti-asexual stage antibodies during pregnancy. 
IgG1, IgG2, IgG3, and IgM Ab concentrations (pg/mL, median and range) against the AMA1, GLURP-R0, GLURP-R2, MSP1, MSP2-3D7, MSP2-FC27 and MSP3 antigens during pregnancy without IPTp intake (at ANV1), after an IPTp intake (at ANV2) and after twice taking IPTp (at delivery). The analyses were carried out using a mixed model and the pairwise comparisons between the two time points were assessed using paired Tukey's method. The Significant p-value is (p < 0.05). Levels of nominal p-values are indicated by: p < 0.1; *p < 0.05; ** p < 0.01; ***p< 0.001. Colored dots in each column indicate individual data points and their distribution.
Supplementary Figure 2. Correlation between anti-asexual stage antibody concentrations in mothers at delivery and in newborns cord blood. 
The figure presents the correlations (r = Pearson's correlation coefficient) between anti-asexual stage Ab concentrations. r=0.60-0.79 as strong correlations and r>0.80 as very strong correlations. Data are log-transformed. Correlation of IgM concentration is not presented because it is known that there is no IgM transfer from the mother to the newborn.
